# New Peptide-Conjugated Chlorin-Type Photosensitizer Targeting Neuropilin-1 for Anti-Vascular Targeted Photodynamic Therapy

**DOI:** 10.3390/ijms161024059

**Published:** 2015-10-12

**Authors:** Ezatul Ezleen Kamarulzaman, Amirah Mohd Gazzali, Samir Acherar, Céline Frochot, Muriel Barberi-Heyob, Cédric Boura, Patrick Chaimbault, Estelle Sibille, Habibah A. Wahab, Régis Vanderesse

**Affiliations:** 1 LCPM UMR 7375, CNRS, ENSIC, 1 rue Grandville, BP 20451-54001 Nancy Cedex, France; E-Mails: ezatulezleen@gmail.com (E.E.K.); amirah.mohd-gazzali@univ-lorraine.fr (A.M.G.); samir.acherar@univ-lorraine.fr (S.A.); 2 LCPM, UMR 7375, Université de Lorraine, ENSIC, 1 rue Grandville, BP 20451-54001 Nancy Cedex, France; 3 School of Pharmaceutical Sciences, Universiti Sains Malaysia, 11800 Penang, Malaysia; E-Mail: bibwahab@gmail.com; 4 LRGP, UMR 7274, CNRS, ENSIC, 1 rue Grandville, BP 20451-54001 Nancy Cedex, France; E-Mail: celine.frochot@univ-lorraine.fr; 5 LRGP, UMR 7274, Université de Lorraine, ENSIC, 1 rue Grandville, BP 20451-54001 Nancy cedex, France; 6 CRAN, UMR 7039, Université de Lorraine, Campus Sciences, BP 70239-54506 Vandœuvre Cedex, France; E-Mails: muriel.barberi@univ-lorraine.fr (M.B.-H.); cedric.boura@univ-lorraine.fr (C.B.); 7 CRAN, UMR 7039, CNRS, Campus Sciences, BP 70239-54506 Vandœuvre Cedex, France; 8 SRSMC, UMR 7565 ICPM, Université de Lorraine, 1 boulevard Arago, 57078 Metz Cedex 3, France; E-Mail: patrick.chaimbault@univ-lorraine.fr; 9 SRSMC, UMR 7565 ICPM, CNRS, 1 boulevard Arago, 57078 Metz Cedex 3, France; 10 LCP-A2MC, EA 4632, ICPM, 1 boulevard Arago, 57078 Metz Cedex 3, France; E-Mail: estelle.sibille@dijon.inra.fr

**Keywords:** photodynamic therapy, peptide targeted photosensitizer, neuropilin-1, *in vivo* stability

## Abstract

Photodynamic therapy (PDT) is a cancer treatment modality that requires three components, namely light, dioxygen and a photosensitizing agent. After light excitation, the photosensitizer (PS) in its excited state transfers its energy to oxygen, which leads to photooxidation reactions. In order to improve the selectivity of the treatment, research has focused on the design of PS covalently attached to a tumor-targeting moiety. In this paper, we describe the synthesis and the physico-chemical and photophysical properties of six new peptide-conjugated photosensitizers designed for targeting the neuropilin-1 (NRP-1) receptor. We chose a TPC (5-(4-carboxyphenyl)-10,15, 20-triphenyl chlorine as photosensitizer, coupled via three different spacers (aminohexanoic acid, 1-amino-3,6-dioxaoctanoic acid, and 1-amino-9-aza-3,6,12,15-tetraoxa-10-on-heptadecanoic acid) to two different peptides (DKPPR and TKPRR). The affinity towards the NRP-1 receptor of the conjugated chlorins was evaluated along with *in vitro* and *in vivo* stability levels. The tissue concentration of the TPC-conjugates in animal model shows good distribution, especially for the DKPPR conjugates. The novel peptide–PS conjugates proposed in this study were proven to have potential to be further developed as future NRP-1 targeting photodynamic therapy agent.

## 1. Introduction

Photodynamic therapy (PDT) is a cancer treatment modality that was discovered many decades ago. The first patients were treated in 1975 by Dougherty *et al.* [1], who successfully eradicated skin cancer with a hematoporphyrin derivative (HpD) in 98 out of 113 patients.

PDT requires three components: light, dioxygen and a photosensitizing agent. Ideally, the photosensitizer (PS) should selectively accumulate into tumor tissue. In reality, relative selectivity is observed due to leaky vasculature, acidic pH of the tumor and also high metabolism of tumor cells. Increasing research is focusing on the design of third generation of PS, which consists of a PS covalently attached to a tumor-targeting moiety or encapsulated within nanoparticles. These tumor-targeting moieties could be biomolecules such as peptides [2,3], monosaccharides [4], low-density lipoprotein (LDL) [5,6] or antibodies [7,8,9], for example. Furthermore, PS can play another major role in the treatment of cancers. In fact, after preferential uptake by malignant cells, PS can act as image-guidance diagnostic tool due to their emission in the near-infrared [10].

Peptides are an attractive choice for targeting strategies because of their small size and ease of synthesis [11]. They have become of interest as tumor-targeting molecules in the field of photodynamic therapy, especially in improving the selectivity of PS towards tumor tissues or neo-vessels [12,13,14]. Active targeting using peptides was found to be useful in helping the delivery of substances [15]. Highly expressed enzyme receptors in cancer cells such as the matrix metalloproteinase enzymes (MMP) [16] and vascular endothelial growth factor receptors (VEGFR) [17] are examples of useful targets.

The peptide-conjugated PS first developed in our group was TPC–Ahx–ATWLPPR. This conjugate consisted of a PS (5-(4-carboxyphenyl)-10,15,20-triphenyl chlorin (TPC)), a spacer (6-aminohexanoic acid (Ahx)) and a peptide sequence (ATWLPPR) [12,13,18,19], which was designed to target the neuropilin-1 receptor (NRP-1), a receptor of vascular endothelial growth factor 165 (VEGF_165_). The conjugate successfully demonstrated enhanced uptake and comparable photodynamic properties to those of the non-conjugated TPC [12], which indicated that the selectivity of PS accumulation in tumor tissue could be enhanced by conjugation with peptide moieties. However, further studies found that this conjugate was degraded *in vivo* into TPC–Ahx–A, leading to loss of selectivity of the peptide moiety [14].

In a submitted paper, we report the binding affinity of nine peptides for NRP-1 [20]. We first performed molecular docking studies to predict the binding interactions of selected peptides with the targeted NRP-1 and subsequently confirmed the molecular affinity *in vitro* by performing competitive binding experiments (ELISA) using recombinant NRP-1 protein. Among the peptides tested, only five were able to displace the binding of VEGF_165_, a physiological ligand of the NRP-1 receptor. These were DKPRR, DKPPR, TKPRR, TKPPR and CDKPRR. Three of these peptides (TKPPR [21], DKPRR [22] and CDKPRR [23]) have already been described in previous works. Two novel pentapeptides DKPPR and TKPRR were chosen to be further conjugated with a chlorin molecule. To this aim, three different spacers were used: Ahx (1-aminohexanoic acid), PEG9 (1-amino-3,6-dioxaoctanoic acid) and PEG18 (1-amino-9-aza-3,6,12,15-tetraoxa-10-on-heptadecanoic acid). These spacers were selected to characterize the influence of spacer lengths and hydrophobicity on the peptides’ affinity towards NRP-1 receptor, as well as on the conjugates’ solubility and polarity profiles. In this paper we report on the *in vitro* conjugates’ competitive binding study and on the *in vivo* stability profiles and make a comparison with results obtained for our previous TPC–Ahx–ATWLPPR conjugate.

## 2. Results

### 2.1. Synthesis of Novel TPC (5-(4-Carboxyphenyl)-10,15,20-triphenyl chlorin)–Peptide Conjugates

Several conjugating strategies between peptides and PS have been suggested with the aim of increasing the accumulation of PS in targeted tumor tissues [12,24,25,26,27,28]. These include direct conjugation between peptide and PS [12,24,25,29,30] conjugating the peptides to a polymer scaffold to which the PS molecules are attached to, or modifying liposome-encapsulating PS with peptides [27]. These conjugation strategies could be performed on either the solid phase peptide synthesis (SPPS) or classical homogenous solution method (liquid phase synthesis) [29].

**Figure 1:**
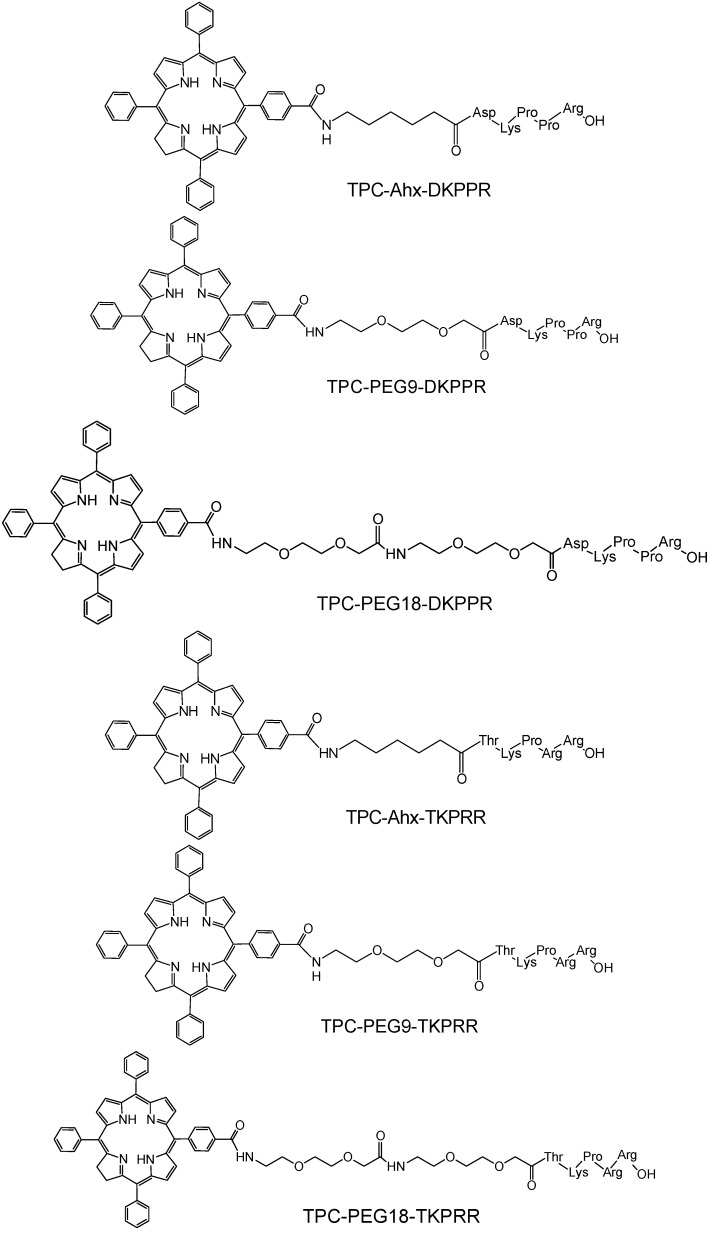
Chemical structures of peptide–spacer–TPC (5-(4-carboxyphenyl)-10,15,20-triphenyl chlorin) conjugates.

In this study, peptides were synthesized on a multi-channel peptide synthesizer with this step followed by the attachment of spacers (Ahx, PEG9 and PEG18, where 9 and 18 refer to the number of atoms of the chain of polyethyleneglycol) and finally the coupling of PS. The coupling by solid phase method allowed site-specific conjugation of PS to the amino-terminal of the peptides in a one to one ratio. The conjugates were cleaved from the resin at the end of the coupling reaction and gave approximately 20%–50% yield after purification. The identities of TPC–peptide conjugates (Figure 1) were well characterized by mass spectrometry (Table 1) and Proton Nuclear Magnetic Resonance (^1^H NMR) spectroscopy. Mass chromatograms and NMR attributions are given (Figure S1 and S2, Table S1–S3) in “Supplementary Information” Section.

**Table 1:** Mass Spectrometry-Electron Spray Ionization (MS-ESI) values of TPC–peptide conjugates.

Conjugate	[M + 2H]^2+^		[M + 3H]^3+^	
	Mass Theory	Experimental	Mass Theory	Experimental
TPC-Ahx-DKPPR	684.8	685	456.9	457
TPC-Peg9-DKPPR	700.8	700	467.5	467
TPC-PEG18-DKPPR *	772.9	773	515.6	516
TPC-Ahx-TKPRR	707.3	707	471.9	472
TPC-Peg9-TKPRR	723.3	723	482.6	482
TPC-Peg18-TKPRR *	795.9	795	530.9	530

* MALDI-TOF spectra are available in “Supplementary Information”.

### 2.2. Distribution Coefficient

The distribution coefficient values at pH 7.4 from the shake flask method and the retention time from HPLC analysis of TPC and the conjugates are summarized in Table 2. The TPC-spacer-peptides were analyzed by HPLC (column Altech, Apollo C18 reverse-phase (5 μm; 250 mm × 4.6 mm)) on a Shimadzu LC-10ATvp, monitored by a UV/Visible detector at 214 and 415 nm on a SPD-10A UV-Visible detector. The octanol/PBS_pH7.4_ distribution coefficient, log D showed significant differences between TPC and the TPC–peptide conjugates, which could be due to the presence of spacers and peptides. The hydrophilicity was found to increase in the following order: Ahx < PEG9 < PEG18. The log *D*_pH7.4_ value for TPC–Ahx–ATWLPPR obtained (2.61 ± 0.2) was also consistent with data published by Tirand *et al.* [13], confirming the hydrophobic characteristic of TPC and the TPC–Ahx–ATWLPPR conjugate.

**Table 2:** Log D values and retention time of TPC and the respective conjugates in reverse phase HPLC.

Sample	Shake Flask Method	Polarity (Methanol/Water), (min)		
	Distribution Coefficient, D	Peak 1 ^a^	Peak 2 ^b^	Average
TPC	2.61 ± 0.2	23.88	24.61	24.18
TPC–Ahx–ATWLPPR	2.61 ± 0.2	23.66	24.41	24.05
TPC–Ahx–DKPPR	1.00 ± 0.2	18.75	19.74	19.35
TPC–PEG9–DKPPR	0.80 ± 0.2	18.53	19.57	19.05
TPC–PEG18–DKPPR	0.31 ± 0.2	17.41	18.41	18.02
TPC–Ahx–TKPRR	0.39 ± 0.2	16.77	17.76	17.41
TPC–PEG9–TKPRR	0.01 ± 0.2	16.68	17.72	17.37
TPC–PEG18–TKPRR	−0.38 ± 0.2	16.29	17.33	16.94

^a,b^ The retention times of the two peaks correspond to the two regioisomers of TPC and TPC–peptide conjugates.

**Figure 2:**
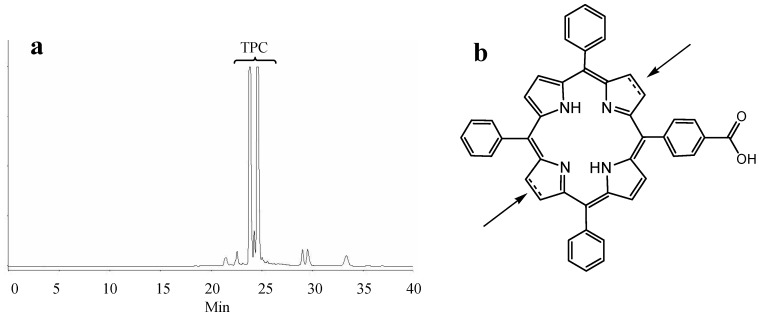
(**a**) HPLC chromatogram of crude TPC at 415 nm; (**b**) The two possible positions of the reduced bond.

As expected, the polarity measurement data of the conjugates as measured by reverse phase HPLC also showed that the conjugation of peptides with TPC via the three different spacers modifies the polarity of TPC. The retention time of the conjugates is inversely proportionate to their polarity; the conjugates with PEG18 were eluted earlier and were therefore more polar than the other conjugates. Hence, the spacer polarity was found to increase in the following order: Ahx < PEG9 < PEG18, which is in accordance with the distribution coefficient study. The DKPPR-based conjugates were found to be less polar than the TKPRR-based conjugates as their longer retention time shows. It is noteworthy that TPC and the TPC conjugates are mixtures of regioisomers (Figure 2).

### 2.3. Photophysical Characterization of Novel Peptides–TPC Conjugates

**Figure 3:**
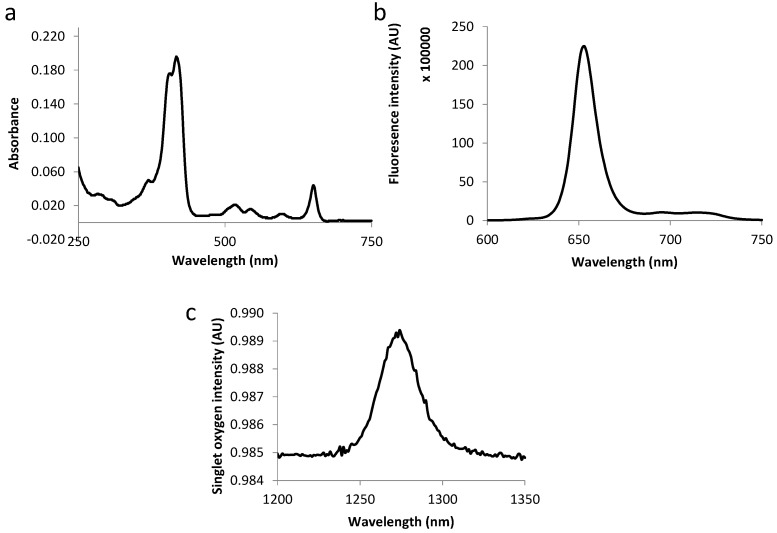
Absorption spectra (**a**), fluorescence emission (**b**) and singlet oxygen emission spectra (**c**) for TPC–PEG18–DKPPR in ethanol.

**Figure 4:**
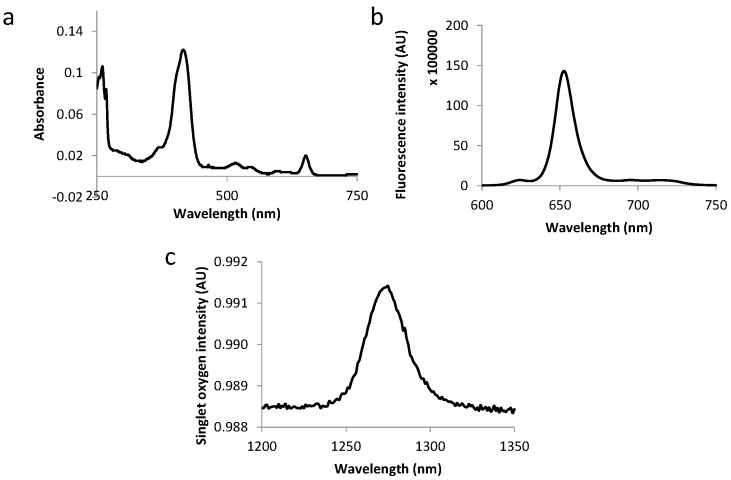
Absorption spectra (**a**), fluorescence emission (**b**) and singlet oxygen emission spectra (**c**) for TPC–PEG9–TKPRR in ethanol.

The fluorescence emission spectra of all compounds displayed two bands at around 650 and 720 nm as expected for chlorins (as exemplified in Figure 3 and Figure 4). Table 3 summarizes the molar extinction coefficient values of the six conjugates as well as their fluorescence and singlet oxygen quantum yield values. No significant variations were observed in conjugates’ photophysical properties as compared with those of free TPC, which complies with the results of other studies [12,18].

**Table 3:** Molar extinction coefficients of TPC and novel TPC–peptide conjugates.

Compound	Molar Extinction Coefficient (L·M^−1^·cm^−1^)					Fluorescence and Singlet Oxygen Quantum Yields	
	Soret	QIV	QIII	QII	QI		
	ε_415_	ε_516_	ε_543_	ε_598_	ε_650_	*Φ_f_*	*Φ_∆_*
TPC	76.00	8.00	5.00	4.00	12.00	0.18	0.47
TPC–Ahx–ATWLPPR	74.00	6.80	4.90	2.60	12.00	0.18	0.39
TPC–Ahx–DKPPR	68.00	6.60	4.90	2.80	14.00	0.23	0.68
TPC–PEG9–DKPPR	92.80	7.40	5.60	3.20	13.00	0.30	0.70
TPC–PEG18–DKPPR	62.40	6.40	4.70	2.80	13.20	0.30	0.69
TPC–Ahx–TKPRR	73.00	4.50	3.50	2.20	10.00	0.22	0.70
TPC–PEG9–TKPRR	79.00	6.40	4.50	2.80	13.90	0.25	0.67
TPC–PEG18–TKPRR	88.30	6.20	4.30	2.40	14.90	0.27	0.67

Molar extinction coefficients determined at maximum wavelengths (ε)_λ_, fluorescence quantum yields (±0.02) (*Φ_f_*) and singlet oxygen quantum yields (±0.05) *Φ_∆_* of the conjugates. All measurements were performed at room temperature in ethanol. TPP solution in toluene was used as the fluorescence standard reference (*Φ_f_*_(*ref*)_ = 0.11) [24] while rose Bengal in ethanol was used as the standard reference for singlet oxygen quantum yields(*Φ_∆_*_(*ref*)_ = 0.68) [25].

### 2.4. Binding Study of Novel TPC–Peptide Conjugates to Recombinant NRP-1 (Neuropilin-1 Receptor) Protein

**Figure 5:**
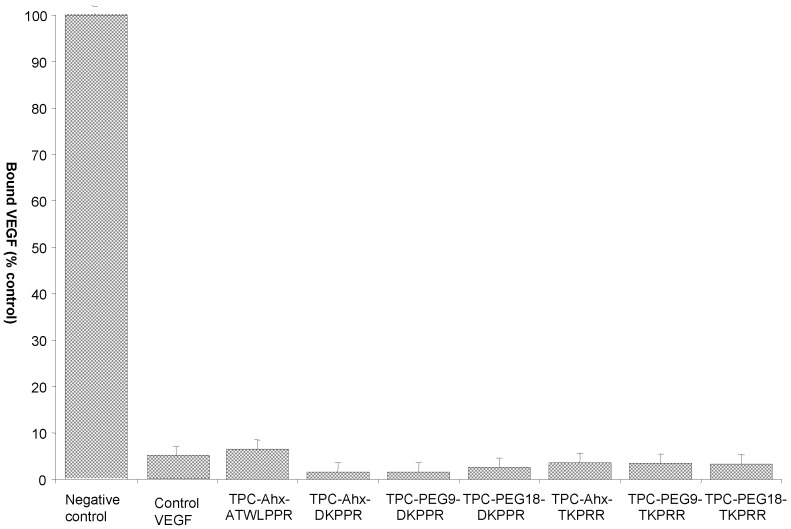
Binding of biotinylated VEGF (vascular endothelial growth) to NRP-1 (neuropilin-1 receptor) in the presence of 2 µg/mL heparin was evaluated in the absence (negative control), or presence, of novel TPC–peptide conjugates to recombinant NRP-1 protein (data points show the mean ± S.D., *n* = 3).

**Figure 6:**
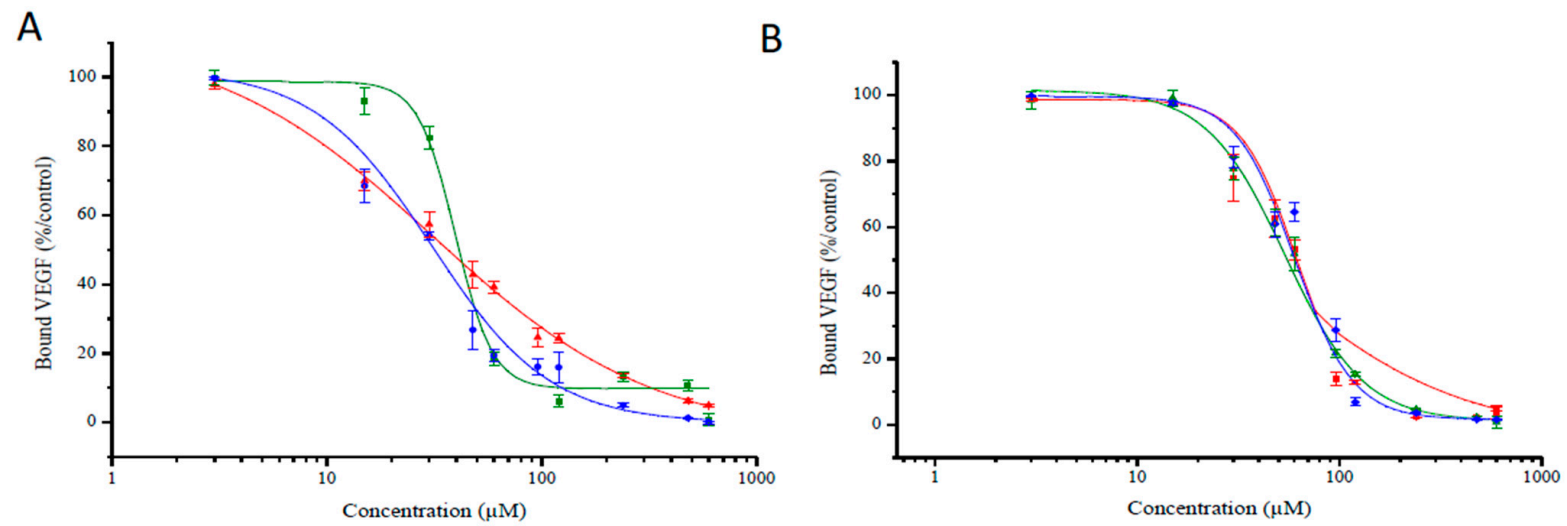
Binding of biotinylated VEGF_165_ (5 ng/mL) to NRP-1 was evaluated at increasing concentration of TPC–peptide conjugates (3 to 960 µM) (data points show the mean ± S.D., *n* = 3). (**A**) Binding of different DKPPR–TPC conjugates to NRP-1 receptor; (**B**) Binding of different TKPRR–TPC conjugates with NRP-1 receptor. The different spacers were illustrated by colors: red for Ahx, green for PEG9 and blue for PEG18.

The molecular affinity of the different TPC–peptide conjugates for recombinant NRP-1 protein was estimated through a competitive binding test using biotinylated VEGF_165_ to NRP-1 receptor recognition recombinant. As biotinylated VEGF_165_ binding to this receptor is heparin dependent, the competitive binding tests were performed in the presence of heparin (2 µg/mL). As expected, free TPC molecules failed to displace the binding of biotinylated VEGF_165_ onto the NRP-1 protein, while the free peptides DKPPR and TKPRR displaced biotinylated VEGF_165_ with an IC_50_ = 1.0 µM (7 μM for ATWLPPR [12]). The TPC–peptide conjugates were found to successfully displace biotinylated VEGF_165_ (Figure 5). For inhibition concentration, IC_50_ (half maximal inhibitory concentration) values of the novel peptides-conjugated chlorin were also assessed and the corresponding values are presented in Figure 6 and Table 4. The IC_50_ values of the conjugates were found to be lower than the previously synthesized TPC–Ahx–ATWLPPR conjugate (IC_50_ = 171 µM) [12], indicating a better affinity for these new conjugated photosensitizers.

**Table 4:** IC_50_ (half maximal inhibitory concentration) values.

Inhibitory Concentration, Mean IC_50_ Values(μM)						
ATWLPPR	DKPPR			TKPRR		
TPC–Ahx–ATWLPPR	TPC–Ahx–DKPPR	TPC–PEG9–DKPPR	TPC–PEG18–DKPPR	TPC–Ahx–TKPRR	TPC–PEG9–TKPRR	TPC–PEG18–TKPRR
171	33	39	30	55	51	53

### 2.5. In Vivo Distribution in Plasma

The *in vivo* stability study was carried out with only two of the novel conjugates synthesized; TPC–PEG18–DKPPR and TPC–PEG18–TKPRR were selected because of to their interesting hydrophilicity properties. We selected TPC–Ahx–ATWLPPR conjugate as a reference based on our previous studies [13]. Blood and tissue samples were weighed and kept at −80 °C in polypropylene tubes, until further processing. Extraction of the conjugates from the tissues required first a solubilization step, using TEM buffer (10 mM Tris, 1.66 mM EDTA, 5 mM molybdate, pH 7.4) and homogenization. Then, the procedure for both plasma and tissue extracts preparation was carried out as described previously [13], with slight modifications, and involved solvent precipitation using methanol combined with DMSO (5:0.1, *v*/*v*). Conjugates levels in the plasma and tissues were determined as a percentage of the injected dose per gram tumor (% injected dose/g of tissue) or per milliliter plasma.

**Figure 7:**
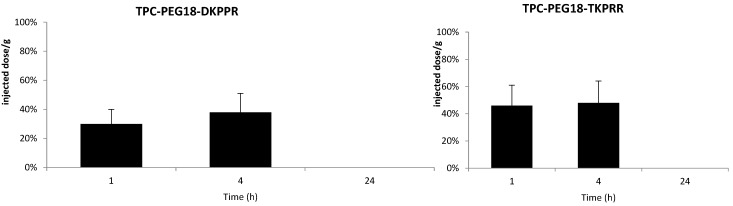
*In vivo* plasma concentrations of TPC–PEG18–DKPPR and TPC–PEG18–TKPRR in mice. The concentrations were determined by HPLC analysis and expressed as a percentage of the injected dose per gram of plasma, and as a function of time at one, four and 24 h after the intravenous injection of 2.8 mg/kg (data points show the mean ± S.D., *n* ≥ 3).

The plasma concentrations of TPC–PEG18–DKPPR and TPC–PEG18–TKPRR following one, four and 24 h after intravenous injection are illustrated on Figure 7. The TPC–Ahx–ATWLPPR conjugate was detected in the plasma up to 24 h after injection. This is consistent with the data reported by Thomas *et al.* [19]. On the contrary, both new conjugates TPC–PEG18–DKPPR and TPC–PEG18–TKPRR were only found in the plasma up to four hours post-intravenous injection, which is probably related to their greater hydrophilicity properties as compared with TPC–Ahx–ATWLPPR.

### 2.6. Tissue Distribution of TPC Conjugates

**Figure 8:**
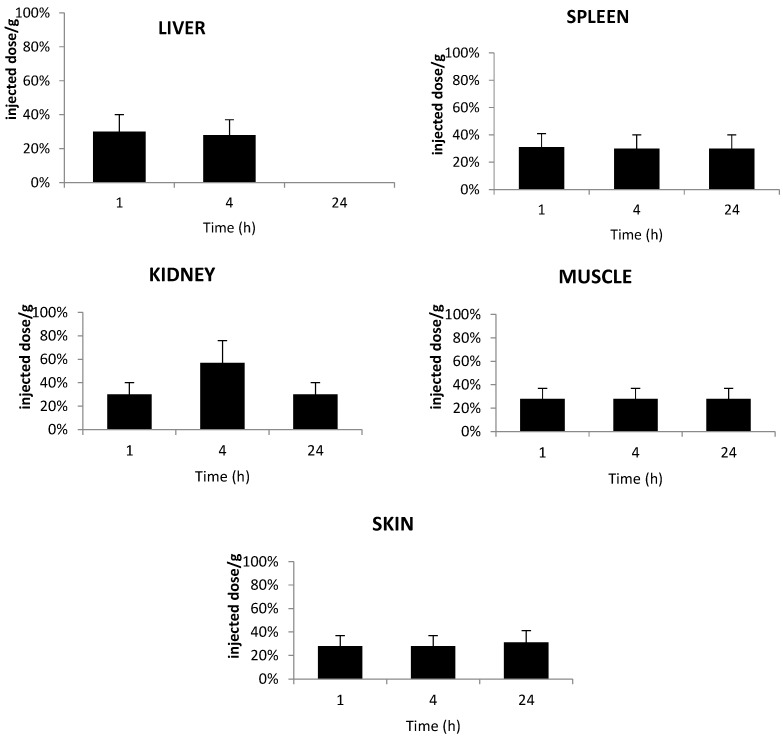
*In vivo* concentrations of TPC–PEG18–DKPPR in the liver, kidneys, spleen, muscle and skin of healthy mice. Concentrations were determined by HPLC analysis and were expressed as the percentage of injected dose per gram plasma at one, 4 and 24 h after the intravenous injection of 2.8 mg/kg (data points show the mean ± S.D., *n* ≥ 3).

**Figure 9:**
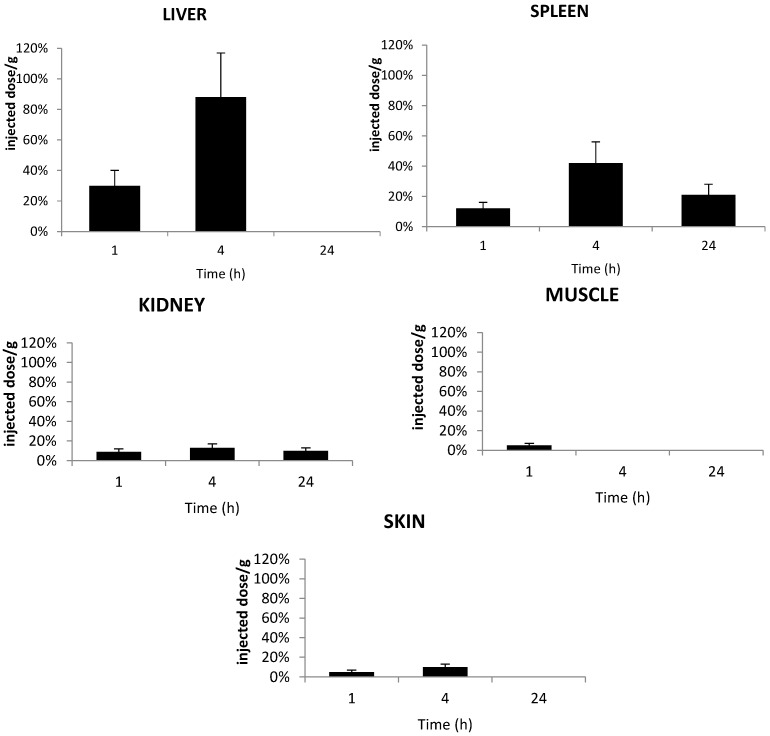
*In vivo* concentrations of TPC–PEG18–TKPRR in the liver, kidneys, spleen, muscle and skin of healthy mice. Concentrations were determined by HPLC analysis of the tested tissues and expressed as the percentage of injected dose per gram plasma, expressed as a function of time at one, four and 24 h after the intravenous injection of 2.8 mg/kg (data points show the mean ± S.D., *n* ≥ 3).

The distributions of TPC–PEG18-DKPPR and TPC–PEG18–TKPRR in healthy mice were determined in different organs: liver, spleen, kidneys, muscle and skin from at least three mice at one, four and 24 h after intravenous injection. The concentrations of the conjugates in each tissue were analyzed by HPLC (Figure 8 and Figure 9).

Figure 8 shows the accumulation of TPC–PEG18–DKPPR in the selected tissues. This conjugate was detected in all tissues until 24 h post-injection apart from the liver at 24 h. This could be explained by the fact that TPC–PEG18–DKPPR is more hydrophilic compared to TPC–Ahx–ATWLPPR (log *D*_pH7.4_ = 0.31 ± 0.2 *versus* log *D*_pH7.4_ = 2.61 ± 0.2).

Figure 9 shows the accumulation of TPC–PEG18–TKPRR in the tested tissues. The conjugate described high accumulation in liver at four hours post-injection, with more than 0.8% of the injected dose per gram tissue found in the liver. TPC–PEG18–TKPRR was found with minimal percentage in the peripheral skin and muscle tissues at one hour post-injection (only 0.05% of injected dose per gram tissue), and its presence was found remain until up to four hours post-injection.

### 2.7. Stability of Conjugates in Plasma and Liver

**Figure 10:**
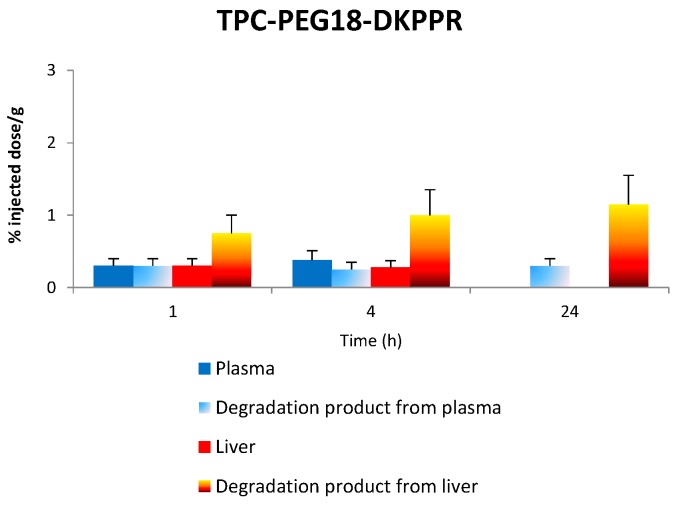

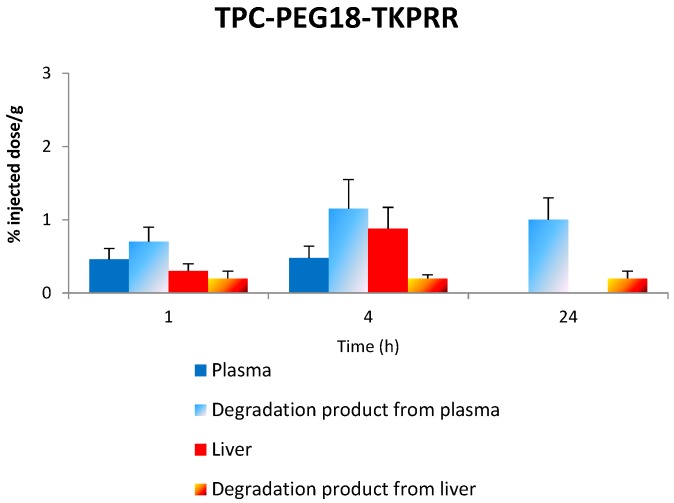
The concentration of the respective conjugates in the plasma and liver along with the concentration of degradation products in both plasma and liver at 1, 4 and 24 h.

Figure 10 gives the *in vivo* concentration of TPC–PEG18–DKPPR and TPC–PEG18–TKPRR in the plasma and liver as well as degraded compounds.

#### 2.7.1. Stability of TPC–PEG18–DKPPR in the Plasma

TPC-PEG18-DKPPR is still persistent in the plasma one hour after its administration (signal is 2500). Thereafter, the peaks observed corresponding to the administration of TPC–PEG18–DKPPR are: *m*/*z* 616: whereas this ion was only observed one hour after administration of TPC–Ahx–ATWLPPR (not detected for 4 and 24 h), it appeared to a significantly degree after 24 h for TPC–PEG18–DKPPR (signal up to 2600). This peak can be attributed to the TPC moiety. (See Figures S3 and S4 in “Supplementary Information”) *m*/*z* 1605 and 1707: Both peaks exhibited a higher mass than the parent drug. The peak at *m*/*z* 1707 was found to appear after 1 h (intensity 480), was still present at four hours (intensity 450) and had disappeared after 24 h. The peak at *m*/*z* 1605 was only present after four hours (intensity 80). Their mass difference compared to the parent drug were + 60 u and + 162 u, respectively. An increase of +162 u often corresponded to glycosylated metabolites (e.g., glucose or other hexose/MW = 180 − 18 = 162/−18 is due to the loss of water during the reaction).

At 24 h, peaks at *m*/*z* 386 and 480 were also detected but they could not be attributed to the degradation of the photosensitizing agent.

#### 2.7.2. Stability of TPC–PEG18–DKPPR in the Liver

The main peaks observed in liver samples were listed in Table 5. All of these can be interpreted on the basis of a hydrolysis of the peptide moiety of the starting molecule (as described for TPC–Ahx–ATWLPPR [13]).

**Table 5:** Mass spectrometry (MS) peaks related to the hydrolysis of TPC–PEG18–DKPPR observed in the liver after 1, 4 and 24 h and their hypothesis of structure (See Figure S4 in “Supplementary Information”).

Observed *m*/*z* *	Hypothesis	Formula **	Theoretical *m*/*z* *	Signal Intensity		
				1 h	4 h	24 h
951.433	TPC–PEG18	C_57_H_55_N_6_O_8_	951.408	Nd ***	200	2000
1066.457	TPC–PEG18–D	C_61_H_60_N_7_O_11_	1066.435	500	1300	200

* Calculated for monoisotopic [M + H]^+^; ** Formula of the protonated molecule; *** not detected.

The Matrix Assisted Laser Desorption/Ionization-Time of Flight Mass Spectrometry (MALDY-TOFMS) signals show that the main cleavage occurs at an early stage of the kinetic (from 1 h on) between aspartic acid (D) and lysine (K) in the peptide moiety. TPC–PEG18 appeared from 4 h on and its detection was unambiguous at 24 h (See Figures S3 and S5 in “Supplementary Information).

#### 2.7.3. Stability of TPC–PEG18–TKPRR in the Plasma

TPC–PEG18–TKPRR was found to behave somewhat like TPC–PEG18–DKPRR. At first, the only peak observed corresponding to the administration of TPC–PEG18–TKPRR was at *m*/*z* 616 and it significantly appeared after 24 h (signal up to 2100). However, MS results also show that TPC–PEG18–TKPRR completely disappeared in the plasma one hour after its administration.

#### 2.7.4. Stability of TPC–PEG18–TKPRR in the Liver

The main peaks observed in liver samples are listed in Table 6. All of them can be interpreted on the basis of a hydrolysis of the peptide moiety of the starting molecule (as described for TPC–Ahx–ATWLPPR and TPC–PEG18–DKPPR).

**Table 6:** MS peaks related to the hydrolysis of TPC–PEG18–TKPRR observed in the liver after 1, 4 and 24 h and their hypothesis of structure (See Figure S5 in “Supplementary Information”).

Observed *m*/*z* *	Hypothesis	Formula **	Theoretical *m*/*z* *	Signal Intensity		
				1 h	4 h	24 h
951.615	TPC–PEG18	C_57_H_55_N_6_O_8_	951.408	200	3400	1200
1052.632	TPC–PEG18–T	C_61_H_62_N_7_O_10_	1052.455	200	3000	Nd ***

* Calculated for monoisotopic [M + H]^+^; ** Formula of the protonated molecule; *** not detected.

The MS signals show that a main cleavage occurs at an early stage of the kinetic (from one hour onwards like for TPC–PEG18–DKPRR) between threonine (T) and lysine (K) in the peptide moiety but also between T and TPC–PEG18 from one hour onwards (See Figures S3 and S6 in “Supplementary Information”).

## 3. Discussion

The synthesis of novel peptides-conjugated photosensitizer started with the synthesis of peptides (DKPPR and TKPRR) using the Fmoc strategy. The spacers (Ahx, PEG9 or PEG18) were then attached to the peptide. Before coupling to the peptide–spacer, TPC was converted into TPC–COOSu and subsequently conjugated to the peptide with DIEA as the coupling agent. This step gave the novel TPC–peptide conjugates TPC–Ahx–DKPPR, TPC–PEG9–DKPPR, TPC–PEG18–DKPPR, TPC–Ahx–TKPRR, TPC–PEG9–TKPRR and TPC–PEG18–TKPRR, which were then purified using reverse phase HPLC to obtain very pure compounds. The HPLC chromatogram produced two peaks, which derive from the isomeric chlorins of TPC.

The photophysical properties of the novel TPC–peptide conjugates were evaluated and the absorption spectra obtained shows the typical chlorin derivatives spectra. This was also in accordance with the findings of Tirand *et al.* [12] and Thomas *et al.* [18]. Not all the conjugated compounds exhibited significant variations in molar extinction coefficients, fluorescence and singlet oxygen quantum yields regardless of the spacers (Ahx, PEG9 or PEG18) and peptides (DKPPR or TKPRR) and in fact showed similar spectra as the non-conjugated TPC.

A competitive binding study comparing the non-conjugated and conjugated TPC showed that conjugated TPC (whether conjugated with TKPRR or DKPPR) succeeded in binding to the NRP-1 receptor while free TPC did not show any significant binding at all. This is indeed in agreement with one previous study, which showed that TPC–ATWLPPR conjugate had managed to displace the binding of VEGF_165_, whereas the non-conjugated TPC failed to do so [14]. This showed that the conjugation of a PS with a peptide may well increase the targeting of the PS towards cancer cells and hence improve the overall photodynamic therapy performance levels. Another study found that the accumulation of TPC–Ahx–ATWLPPR conjugate in NRP-1 and KDR-expressing HUVEC was higher than for non-conjugated TPC [12]. We previously demonstrated that TPC–Ahx–ATWLPPR was a much more potent photosensitizer *in vitro* than TPC, in NRP-1-expressing cells [13]. Indeed, for *in vitro* experiments, human umbilical vein endothelial cells (HUVEC), pooled from several donors, were used and were incubated with either of the photoactive compounds (TPC or TPC–Ahx–ATWLPPR) and irradiated by red light. Whereas the control photosensitizer TPC displayed little photodynamic activity in HUVEC, conjugation with ATWLPPR significantly enhanced photodynamic activity (10.4-fold). Light doses 50 values, after incubation with either TPC–Ahx–ATWLPPR or TPC, were 0.47 ± 0.23 and 4.9 ± 0.64 J/cm^2^, respectively. This wide difference in photocytotoxicity was consistent with the dissimilar accumulation of the photoactive compounds in HUVEC.

The binding of biotinylated VEGF_165_ to NRP-1 was displaced by the novel conjugates synthesized in this study in a concentration-dependent manner. This showed that the spacer length and its nature have no influence on the affinity of the peptides towards its receptor but instead the spacers were however found to increase the solubility of the conjugates. The novel conjugates also showed better affinity towards NRP-1 compared with TPC–Ahx–ATWLPPR (IC_50_ = 171 µM [12]).

The conjugated peptides were found to have lower binding affinity than free peptides which could be due to the presence of TPC close to the peptide moiety which may cause steric hindrance and hence difficulty in binding. The aim of using a spacer was to ensure the presence of space between TPC and peptide so that these were individualized and separated. Several studies have reported that the presence of a spacer may bring flexibility to the molecule and that its length as well as the nature of the molecule attached to it may have an impact on receptor affinity [26,27]. In one study, folic acid was conjugated to 4-carboxyphenylporphyrin via two short spacers (hexane-1,6-diamine or 2,2′-(ethylenedioxy)-bis-ethylamine), which were different in nature but similar in size [28]. Both conjugated PSs showed improved intracellular uptake and photodynamic activity in human oropharyngeal epidermoid carcinoma KB cells as compared with the non-conjugated PS. This indicates that the type of spacers have no effect on the activity of the conjugated compounds. A study by Tirand *et al.* [12] compared the IC_50_ values of the ATWLPPR peptide with the Ahx–ATWLPPR conjugate and found that there was no significant change on the IC_50_ values (19 and 22 µM, respectively), which demonstrated that the presence of Ahx did not interfere with the binding of the ATWLPPR peptide moiety on the NRP-1 receptor.

*In vivo* studies were performed to characterize the distribution of the conjugates in plasma and in healthy tissues (skin, muscle, liver, kidney and spleen) as a function of time. The main degradation products in plasma and liver were characterized by MALDI-TOF mass spectroscopy and were evaluated after one, four and 24 h after injection. The plasma concentration of the new conjugates TPC–PEG18–DKPPR and TPC–PEG18–TKPRR were significantly different from that of TPC–Ahx–ATWLPPR [13]. While TPC–Ahx–ATWLPPR could be detected in the plasma for 48 h or more, the two new conjugates were found to be present in the plasma for only 4 h. This may be due to the difference of hydrophobicity between Ahx and PEG18 and the nature of the peptides. TPC was observed at one hour after injection of TPC–PEG18–DKPPR. Another peak at *m*/*z* 1605 corresponding to a glycosylated parent drug was also observed. TPC–PEG18–TKPRR behaves in the same way as the DKPPR analogous with a TPC peak, which appears to a significant degree after 24 h.

A tissue distribution study of the TPC–PEG18–DKPPR and TPC–PEG18–TKPRR conjugates showed high accumulation into the organs of the reticulo-endothelial system in comparison with the peripheral muscle and skin tissues. TPC–PEG18–DKPPR was present in all tissues even after 24 h. A maximum concentration was observed in kidney after 4 h. In liver, the main cleavage occurs at an early stage of the kinetic (from 1 h on) between aspartic acid (D) and lysine (K) in the peptide moiety. TPC–PEG18 appears from 4 h on and its detection is unambiguous at 24 h.

TPC–PEG18–TKPRR was found to have a high accumulation in the spleen and even more in the liver. It was almost nonexistent in the kidney, muscle and skin. In liver, the main degradation product after injection is TPC–PEG18–T. Another cleavage between PEG18 and T occurred after four hours and reached a significant level at 24 h.

## 4. Experimental Section

### 4.1. Synthesis of TPC–Peptide Conjugates

5-(4-carboxyphenyl)-10,15,20-triphenylporphyrin was successfully synthesized through modification of the method described by Bonnett *et al.* [29]. It was obtained in the form of a purple solid with a yield of 43%. Subsequently, 5-(4-carboxyphenyl)-10,15,20-triphenylchlorin (TPC) was obtained through diimide reduction via the Whitlock method [30], with a final yield of approximately 25%. TPC was purified on a C18 semi-preparative column (150 × 10 mm, Apollo, Alltech, Lokeren, Belgium) using a 0.1% (*v*/*v*) TFA-water/methanol gradient, with a flow rate of 4.0 mL/min and monitored by absorbance at 415 and 650 nm on a SPD-10A UV-Visible detector (Shimadzu, Marne la Vallée, France). During purification by HPLC, TPC was eluted as double peaks at 23.90 and 24.60 (±1 min), which corresponded to the presence of two isomers of this molecule, due to the asymmetric reduction of double bonds on either one side of the tetrapyrrolic macrocycle. TPC-NHS was prepared in the dark under a nitrogen atmosphere by the action of one equivalent of both *N*-hydroxysuccinimide (NHS) and dicyclohexylcarbodiimide (DCC) in DCM overnight at room temperature. The reaction was monitored by TLC carried out on Merck silica gel 60 F_254_ plates (Merck Chimie S.A.S., Fontenay-sous-Bois, France) and the spots were visualized under UV light. The solvent was evaporated and the crude product was then purified by HPLC on the same column and in the same conditions as TPC.

All peptides were synthesized by using the Fmoc/tBu method with HBTU activation using an automated ResPep XL peptide synthesizer (Intavis AG, Bioanalytical Instruments, Köln, Germany) and operated with a Multiple-Parallel Peptide Synthesis Program. The amounts of reagents are given in equivalents (eq.). Double coupling was carried out by using a 3-fold excess of *N*-Fmoc amino acid and activation reagents 2-(1*H*-benzotriazol-1-yl)-1,1,3, 3-tetramethyl-uronium tetrafluoroborate (HBTU) (3 eq.), 1-hydroxybenzotriazole (HOBt) and *N*,*N*-diisopropylethylamine (DIEA) (9 eq.) in dimethylformamide (DMF). Unless otherwise stated, reagents were purchased from chemical companies and used without prior purification. The resins and Fmoc-amino acids were from Iris Biotech GmbH, Marktredwitz, Germany. The three linkers were then attached with Fmoc-Ahx-OH, Fmoc-PEG9-OH and Fmoc-PEG18-OH like normal amino acids. TPC (1.1 equivalents was then coupled to the N-terminus of the each peptide-spacer on the resin via its NHS ester in a solution of DMF/DCM (1:1, *v*/*v*) and DIEA (9 eq.). The resulting TPC–spacer–peptides were cleaved from the resin by treating with a mixture of trifluoroacetic acid/triisopropylsilane/water (TFA/TIPS/H_2_O; 92.5/5.0/2.5). The resulting crude TPC–spacer–peptides were purified by HPLC (column Altech, Apollo C18 reverse-phase (5 µm; 250 mm × 4.6 mm)) on a Shimadzu LC-10ATvp, monitored by a UV/Visible detector at 214 and 415 nm on a SPD-10A UV-Visible detector (Shimadzu, Marne la Vallée, France). Pure TPC–peptide conjugates obtained were analyzed accordingly by mass spectroscopy and ^1^H NMR, COSY and TOCSY).

### 4.2. Chemical Characterization of TPC and the Novel TPC–Peptide Conjugates

Verification of the relative atomic mass of the conjugates was carried out using Liquid Chromatography Mass Spectrometry (LCMS) on a LCMS-2010 EV (Shimadzu Corporation, Marne-la-Vallée, Paris, France) equipped with a diode array detector SPD-M20A, a column oven CT0-20AC and a DGU-20A3degasser.^1^H NMR spectra were recorded on a Bruker Avance 300 MHz spectrometer (Bruker, Wissenburg, Germany). All samples were prepared in deuterated dimethyl sulfoxide (DMSO-*d*_6_) with a concentration of about 10 mM.

Hydrophilic and hydrophobic characteristics were also evaluated using the “shake flask” method to determine the solubility of the free TPC and the conjugates. The distribution coefficients D of the compounds between the non-polar (octanol) and polar phases (PBS_pH7.4_) were hence determined by using Equation (1):

(1) $$D_{\mathrm{pH7.4}}=\frac{C_{\mathrm{octanol}}}{C_{\mathrm{PBS}}}=\frac{I_{f\mathrm{(octanol)}}}{I_{f\mathrm{(PBS)}}}=$$

where, *C*_octanol_ is the concentration of compound species in octanol, *C*_PBS_ is the concentration of compound species in PBS, while *I_f_*_(octanol)_ and *I_f_*_(PBS)_ are the areas under fluorescence spectra between 600 and 750 nm of the species in octanol and PBS, respectively. The distribution coefficient, *D*_pH7.4_ was then expressed as log (mean ± S.D., *n* = 6).

### 4.3. Photophysical Characterization of Novel TPC–Peptide Conjugates

The photophysical properties of TPC and the TPC–peptide conjugates were characterized using three different techniques; UV/visible absorption (Perkin Elmer Lambda 2 UV-Vis spectrophotometer, Courtaboeuf, France), fluorescence emission (SPEX Fluorolog-3 spectrofluorometer, Jobin Yvon, Longjumeau, Paris, France) and singlet oxygen emission. For UV-Vis measurements, the samples of TPC and the six conjugates were diluted in ethanol and their spectra were determined in the range of 300 to 800 nm. For fluorescence emission spectra, samples with an absorption at 415 nm equal to 0.200 (±0.02) were prepared. Solvent refractive index and absorption efficiencies of fluorescence quantum yields (*Φ_f_*) were calculated based on a TPP solution in toluene as a fluorescence standard reference (*Φ_f_*_(*ref*)_ = 0.11) [24] as described by Equation (2):

(2) $$\phi_{f}=\phi_{f(ref)}\times \frac{I}{I_{ref}}\times \frac{A_{ref}}{A}\times {\left(\frac{n}{n_{ref}}\right)}^{2}$$

where *A* and *A_ref_* correspond to the absorbance at 415 nm of the sample and TPP, respectively, while *I* and *I_ref_* are the areas under the fluorescence spectra between 600 and 750 nm of the sample and TPP, respectively. *n* and *n_ref_* are the refractive indices of solvents used for the sample and TPP, respectively (*n/n_ethanol_* = 1.361, *n_ref_*/*n_toluene_* = 1.496).

Singlet oxygen emission spectra were recorded on SPEX 1680 with 0.22 mm double monochromator with a Xenon arc lamp as excitation source. Singlet oxygen generation was specifically detected at 1270 nm and the emission was monitored by using liquid nitrogen-cooled germanium-detector (EO-817P, North Coast Scientific, North Ridgeville, OH, USA). A solution of rose Bengal in ethanol was used as the reference, *Φ*_Δ(*ref*)_ = 0.68 [25]. The singlet oxygen quantum yield was then determined using Equation (3):

(3) $$\phi_{\Delta }=\phi_{\Delta (ref)}\times \frac{I}{I_{ref}}\times \frac{A_{ref}}{A}$$

where *A* and *A_ref_* are the UV visible absorbance at 415 nm of the sample and rose Bengal (the reference), respectively, while *I* and *I_ref_* are the peak intensities of luminescence at 1270 nm of the sample and rose Bengal, respectively.

### 4.4. Binding of Peptides and Conjugates to Recombinant KDR and NRP-1 Proteins

HUVEC, pooled from several donors, were used (Cambrex, Verviers, Belgium). These cells were routinely grown in endothelial growth medium (EGM-2), containing 2% fetal bovine serum (FBS), growth factors and supplements, and maintained according to the manufacturer’s instructions. Only cells from passages 3–7 were used for our experiments. The binding affinities of the conjugates (TPC–Ahx–DKPPR, TPC–PEG9–DKPPR, TPC–PEG18–DKPPR, TPC–Ahx–TKPRR, TPC–PEG9–TKPRR and TPC–PEG18–TKPRR) were evaluated through the enzyme-linked immunosorbent assay, the ELISA study. The conjugates were first dissolved in DMSO and subsequently diluted in methanol. Tween-20 was added in the blocking buffer to assist in the solubilization of the conjugate. The samples were prepared in ten different concentrations ranging from 3 to 960 µM. Affinities were expressed as IC_50_, which means the concentration of competitor (TPC–peptide conjugates) that has the ability to displace 50% of biotinylated VEGF_165_ binding on the NRP-1 receptor, using the medium effect method.

### 4.5. In Vivo Study

Female athymic Swiss nude/nude mice (7–9 weeks, weight range 20–25 g) were obtained from Harlan (Gannat, France). The *in vivo* study we conducted was based on the results obtained from the ELISA test conducted earlier. The conjugates of TPC–Ahx–ATWLPPR, TPC–PEG18–DKPPR and TPC–PEG18–TKPRR (2.8 mg/kg) were injected intravenously via the tail vein in ethanol–PEG 400–water (2:3:5, *v*/*v*/*v*). The mice were kept in the dark after the injection. After 1, 4 and 24 h, blood samples were collected and plasma was separated from the blood. The mice were sacrificed and tissues were carefully excised. All samples were kept in the dark at −80 °C in polypropylene tubes until further analysis.

#### 4.5.1. Preparation of Samples for HPLC Analysis

The surface blood from the tissues was removed by rinsing in physiological saline, blotted dry and weighed. The tissue samples were then homogenized in 500 µL of Tris–EDTA–molybdate buffer pH 7.4 before being spiked with 100 μL of 5,10,15,20-tetrakis(*m*-hydroxyphenyl) porphyrin (*m*THPP) (500 ng/mL in methanol) as an internal standard. The next step involved solvent precipitation using methanol and DMSO (5:0.1, *v*/*v*).

Samples were then vortexed and homogenized for 30 min followed by sonication for 10 min (Branson 1200, Roucaire Instruments Scientifiques, Les Ulis, France). Tissue and cellular debris were separated by centrifugation (2500× *g*; 15 min). The PS-containing organic phase was concentrated by evaporation with Speedvac apparatus (Fisher Bioblock Scientific, Illkirch, France). The residue was then reconstituted in methanol (200 µL). Calibration curves were prepared by mixing plasma and organs with appropriate concentrations of conjugates in the range of 50–1000 ng/mL. For these control and calibration samples, the conjugate extraction procedure was identical to the method described above.

#### 4.5.2. *In Vivo* Stability Analysis by HPLC Technique of TPC–Ahx–ATWLPPR, TPC–PEG18–DKPPR and TPC–PEG18–TKPRR in Plasma and Tissue Distribution of the Conjugates

The *in vivo* stability analyses were performed by HPLC equipped with programmable software GOLD version 1.6 (Beckman Coulter, Fullerton, CA, USA), an auto sampler injector (507e, System Gold, Beckman Coulter) and a fluorescence detector (RF-10A XL, Shimadzu, Kyoto, Japan). Analyses were performed by RP-HPLC on a C18 analytical column (250 × 4.6 mm, YMC, Interchim, Montluçon, France). Isocratic elution condition was applied with a mobile phase of methanol/water (95:5, *v*/*v*) and a flow rate of 1.0 mL/min. Fluorescence excitation and emission wavelengths were detected at 416 and 652 nm, respectively. All the solvents used were of the analytical grade quality. The conjugate levels in plasma and organs were determined from the peak areas and normalized based on standard calibration curves constructed earlier. The data were calculated as the mean value of three mice.

#### 4.5.3. Statistical Analysis

Unless otherwise indicated, all data are mean values ± standard deviations (S.D.) calculated from at least three independent data experiments.

#### 4.5.4. Mass Spectrometry

MALDI-TOF MS analyses were carried out on a Bruker Reflex IV time-of-flight mass spectrometer (Bruker-Daltonic, Bremen, Germany) equipped with the SCOUT 384 probe ion source fitted with a nitrogen pulsed laser (337 nm, VSD-337ND model, Laser Science Inc., Boston, MA, USA). The laser output energy was 400 μJ/pulse. Positives ions were accelerated with a 200 ns extraction delay. The reflector voltage was 23 kV.

Mass spectra were manually acquired using Flex-control software (Bruker Daltonic, Bremen, Germany) by accumulating four series of 100 laser shots (at 45% of total laser energy).

MALDI-TOF MS analyses were performed using 2,5-dihydroxybenzoic acid (DHB) (Sigma-Aldrich, Saint-Quentin-Fallavier, France) as the matrix. This matrix was prepared at a concentration of 1 M in an acetonitrile-water mixture (50/50, *v*/*v*) acidified with 0.1% trifluoroacetic acid (TFA) (Merck, Darmstadt, Germany). All deposits were carried out using the dried-droplet method with 1 μL of both analyte and matrix. The detection of pure products by MALDI-TOFMS was checked using 10^−4^ M solutions of analyte. External calibration was carried out using DHB matrix peaks and a mixture of standard peptides (Bruker Daltonic, Bremen, Germany). The following monoisotopic peaks were used: *m*/*z* 155.034 ([DHB + H]^+^), *m*/*z* 757.400 (bradikynin), *m*/*z* 1046.542 (human angiotensin II) and *m*/*z* 1533.860 (P_14_R). The calibration was considered successful if the rms error was in the range of ±10–15 ppm. Results were accepted when their rms error were less than 50 ppm. In order to highlight mass peaks resulting from the hydrolysis of the targeted photosensitizers in the different organs after 1, 4 and 24 h, peaks coming from the 2,5-DHB and from the tissue were removed from the list. All samples were analyzed in duplicate.

## 5. Conclusions

In conclusion, the novel TPC–peptide conjugates proposed in this study were proven to have potential for further development as future NRP-1 targeting photodynamic therapy agents. They were found to display good distribution levels in an animal model especially the DKPPR conjugates. It would be interesting to investigate their biodistribution in tumor-bearing mice. More research would need to be done to further evaluate their properties and thus improve the targeting capability of the conjugates.

## Supplementary Materials

Supplementary materials can be found at http://www.mdpi.com/1422-0067/16/10/24059/s1.

## Abbreviations

Ahx: 6-aminohexanoic acid; Arg: Arginine; Asp: Aspartic acid; DCM: dichloromethane; DIEA: *N*,*N*-diisopropylethylamine; DMF: dimethylformamide; DMSO: dimethylsulfoxide; EDTA: ethylenediaminetetraacetic acid; ELISA: enzyme-linked immunosorbent assay; ESI: electrospray ionization; HpD: haematoporphyrin derivative; HPLC: high-performance liquid chromatography; IC: inhibitory concentration; LC-MS: liquid chromatography-mass spectrometry; LDL: low-density lipoprotein; Lys: Lysine; MMP: matrix metalloproteinase; MS: mass spectrometry; *m*THPP: 5,10,15,20-tetrakis(*m*-hydroxyphenyl) porphyrin; NMR : nuclear magnetic resonance; NRP-1: neuropilin-1; PBS: phosphate-buffered saline; PDT: photodynamic therapy; PEG: polyethylene glycol; Pro: Proline; PS: photosensitizer; RP-HPLC: reverse phase-high performance liquid chromatography; SPPS: solid phase peptide synthesis; TFA: trifluoroacetic acid; Thr: Threonine; TPC: 5-(4-carboxyphenyl)-10,15,20-triphenyl chlorine; TPP: triphenylporphyrin; UV: ultraviolet; VEGF: vascular endothelial growth factor; VEGFR: vascular endothelial growth factor receptor.
