# Supplementary material for: New Peptide-Conjugated Chlorin-Type Photosensitizer Targeting Neuropilin-1 for Anti-Vascular Targeted Photodynamic Therapy
Source: Int J Mol Sci. 2015 Oct 12;16(10):24059–80. doi: 10.3390/ijms161024059 (PMC4632738; doi:10.3390/ijms161024059)
Supplement: Supplementary file 1 [file ijms-16-24059-s001.pdf]

Supplementary Information

Table S1. Comparison of the theory and experimental mass for peptides-conjugated TPC (5-(4-carboxyphenyl)-10,15,20-triphenyl chlorine).

Conjugate	$[M + 2H]^{2+}$		$[M + 3H]^{3+}$	
	**Mass Theory (m)**	**Experimental (*m*/*z*)**	**Mass Theory (m)**	**Experimental (*m*/*z*)**
TPC–Ahx–DKPPR	684.8	685	456.9	457
TPC–PEG9–DKPPR	700.8	700	467.5	467
TPC–PEG18–DKPPR	772.9	773	515.6	516
TPC–Ahx–TKPRR	707.3	707	471.9	472
TPC–PEG9–TKPRR	723.3	723	482.6	482
TPC–PEG18–TKPRR	795.9	795	530.9	530

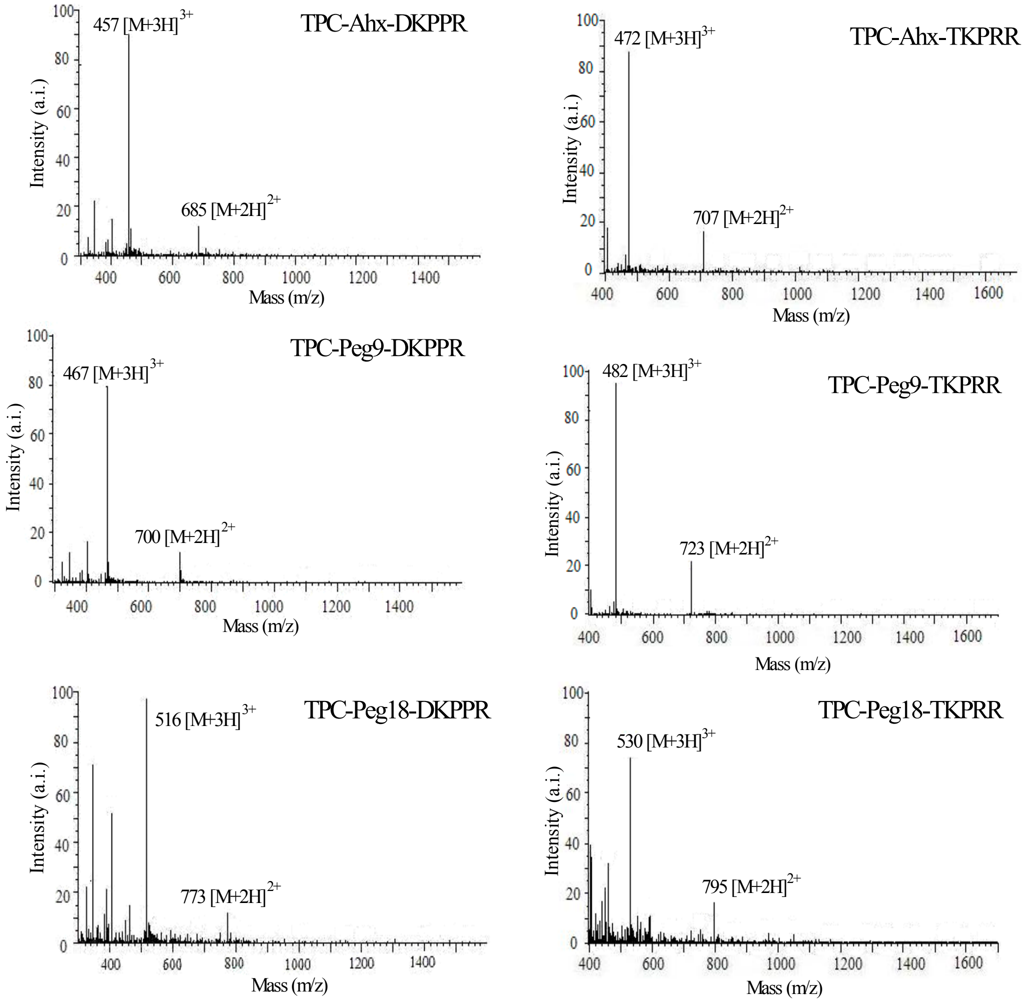

Figure S1. Mass spectra for all conjugates: DKPPR–conjugated TPC (**left**) and TKPRR–conjugated TPC (**right**).

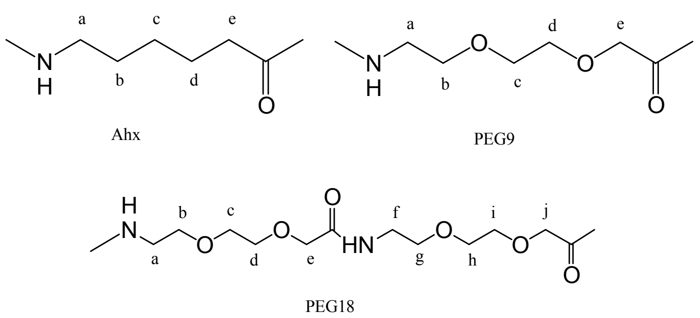

Figure S2. Molecular structures of the spacers used.

Table S2. Chemical shifts of proton NMR (nuclear magnetic resonance) for DKPPR–conjugated TPC: ^{1}H NMR (300 MHz, DMSO-d_6): δ (ppm).

			**TPC–Ahx–DKPPR**			
**Residue**	**NH**	**α-H**	**β-H**	**γ-H**	**δ-H**	**Others ***
TPC	-	-	-	-	-	−1.52, −1.59 (NH-pyrrole); 8.20–8.60 (ArH, pyrrole)
Ahx	8.06	-	-	-	-	a = 3.25; e = 2.15; b, d = 1.44; c = 1.28 *
Asp	8.58	4.38	2.64, 2.75	-	-	-
Lys	8.02	4.50	1.80	1.44	1.70	ε-NH_3^+ = 7.70; ε-CH_2 = 3.38
Pro	-	4.44	2.21	2.06	3.55	-
Pro	-	4.53	2.30	2.10	3.65	-
Arg	8.06	4.34	1.75	1.70	1.80	ε-NH = 7.72
			**TPC–PEG9–DKPPR**			
**Residue**	**NH**	**α-H**	**β-H**	**γ-H**	**δ-H**	**Others ***
TPC	-	-	-	-	-	−1.53, −1.58 (NH-pyrrole); 8.20–8.60 (ArH, pyrrole)
PEG9	8.1	-	-	-	-	e = 4.49; c = 3.67; d = 3.57; b = 3.50; a = 3.36 *
Asp	8.60	4.40	2.66, 2.80	-	-	-
Lys	8.06	4.50	1.73	1.43	1.68	ε-NH_3^+ = 7.65; ε-CH_2 = 3.38
Pro	-	4.47	2.23	2.08	3.55	-
Pro	-	4.55	2.32	2.13	3.65	-
Arg	8.09	4.36	1.80	1.75	1.85	ε-NH = 7.73
			**TPC–PEG18–DKPPR**			
**Residue**	**NH**	**α-H**	**β-H**	**γ-H**	**δ-H**	**Others ***
TPC	-	-	-	-	-	−1.53, −1.58 (NH-pyrrole); 8.20–8.60 (ArH, pyrrole)
PEG18	8.20	-	-	-	-	e, j = 4.47; b, g = 3.57; c, d, h, i = 3.45; a, f = 3.30 *
Asp	8.58	4.33	2.50, 2.66	-	-	-
Lys	8.00	4.60	1.76	1.46	1.72	ε-NH_3^+ = 7.73; ε-CH_2 = 3.34
Pro	-	4.45	2.21	2.07	3.61	-
Pro	-	4.51	2.28	2.12	3.67	-
Arg	8.18	4.35	1.76	1.77	1.87	ε-NH = 7.75

* a–j refer to methylene groups –CH_2– shown in Figure S2.

Table S3. Chemical shifts of proton NMR for TKPRR-conjugated TPC: ^{1}H NMR (300 MHz, DMSO-d_6): δ (ppm).

**TPC–Ahx–TKPRR**						
**Residue**	**NH**	**α-H**	**β-H**	**γ-H**	**δ-H**	**Others ***
TPC	-	-	-	-	-	−1.53, −1.58 (NH-pyrrole); 8.20–8.60 (ArH, pyrrole)
Ahx	8.20	-	-	-	-	a = 3.32; e = 2.21; b, d = 1.44; c = 1.30 *
Thr	8.07	4.33	4.17	1.05	-	-
Lys	8.02	4.55	1.73	1.45	1.74	ε-NH_3^+ = 7.56; ε-CH_2 = 3.33
Pro	-	4.45	2.19	2.04	3.49	-
Arg	7.91	4.35	1.73	1.68	3.60	ε-NH = 7.73
Arg	8.03	4.39	1.76	1.71	3.63	ε-NH = 7.75
**TPC–PEG9–TKPRR**						
**Residue**	**NH**	**α-H**	**β-H**	**γ-H**	**δ-H**	**Others ***
TPC	-	-	-	-	-	−1.53, −1.58 (NH-pyrrole); 8.20–8.60 (ArH, pyrrole)
PEG9	8.20	-	-	-	-	e = 4.50; c = 3.92; d = 3.70; b = 3.50; a = 3.10 *
Thr	8.19	4.45	4.20	1.13	-	-
Lys	8.09	4.56	1.78	1.50	1.75	ε-NH_3^+ = 7.75; ε-CH_2 = 3.40
Pro	-	4.45	2.20	2.05	3.48	-
Arg	8.09	4.34	1.75	1.70	3.62	ε-NH = 7.68
Arg	8.21	4.40	1.77	1.73	3.65	ε-NH = 7.73
**TPC-PEG18-TKPRR**						
**Residue**	**NH**	**α-H**	**β-H**	**γ-H**	**δ-H**	**Others ***
TPC	-	-	-	-	-	−1.53, −1.58 (NH-pyrrole); 8.20–8.60 (ArH, pyrrole)
PEG18	8.10	-	-	-	-	e, j = 4.50; b, g = 3.51; c, d, i, h = 3.44; a, f = 3.22 *
Thr	8.18	4.50	4.13	1.14	-	-
Lys	8.20	4.55	1.79	1.55	1.79	ε-NH_3^+ = 7.73; ε-CH_2 = 3.39
Pro	-	4.50	2.25	2.11	3.55	-
Arg	8.09	4.40	1.77	1.72	3.64	ε-NH = 7.70
Arg	8.18	4.46	1.80	1.75	3.68	ε-NH = 7.65

* a–j refer to methylene groups –CH_2– shown in Figure S2.

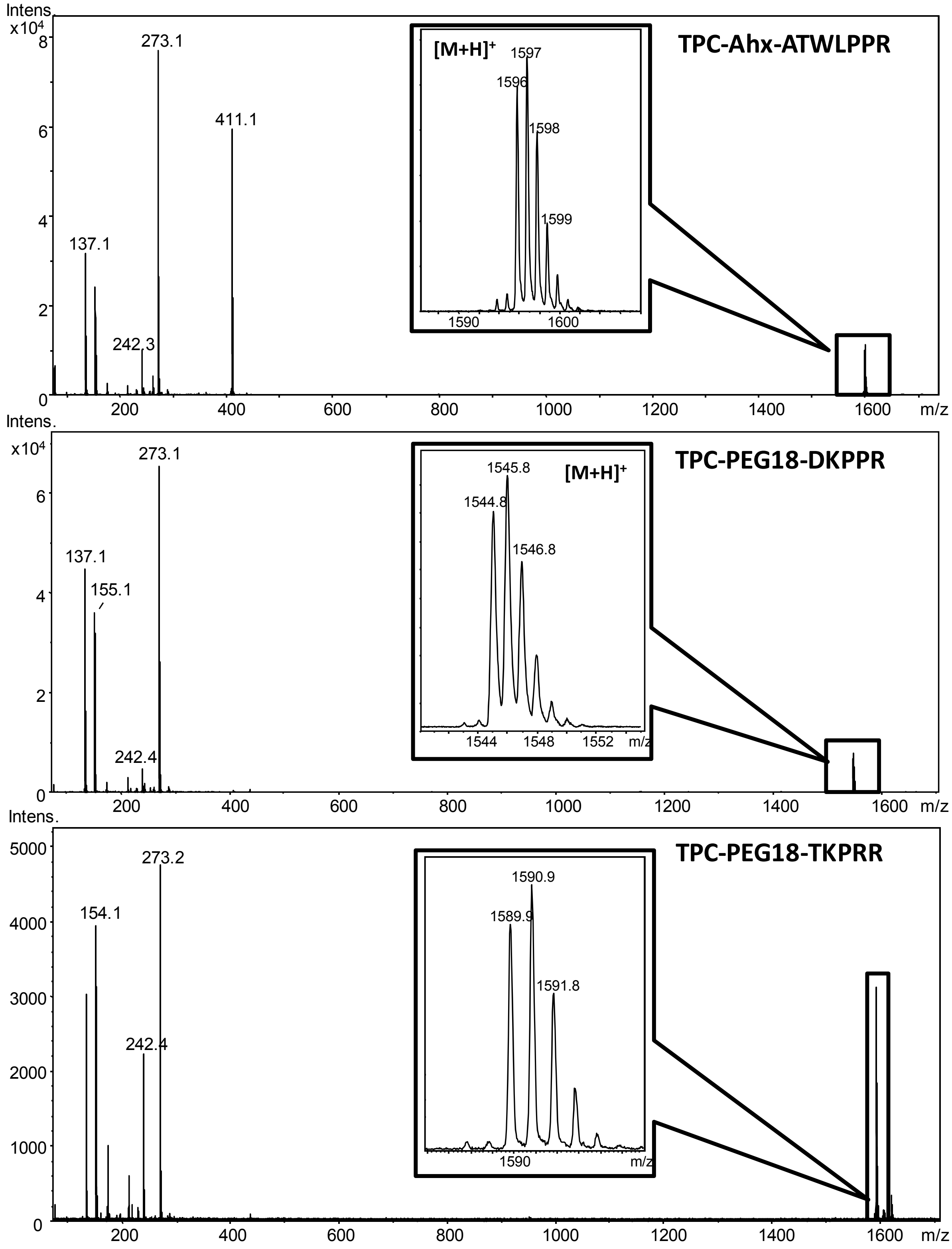

Figure S3. MALDI-TOFMS (Matrix Assisted Laser Desorption/Ionization-Time of Flight Mass Spectrometry (MALDY-TOFMS) spectra of pure products: TPC–Ahx–ATWLPPR; TPC–PEG18–DKPPR and TPC–PEG18–TKPRR (standard). Mass values are given with only one significant digit to avoid overloading the figure. Low mass peaks belong to the matrix.

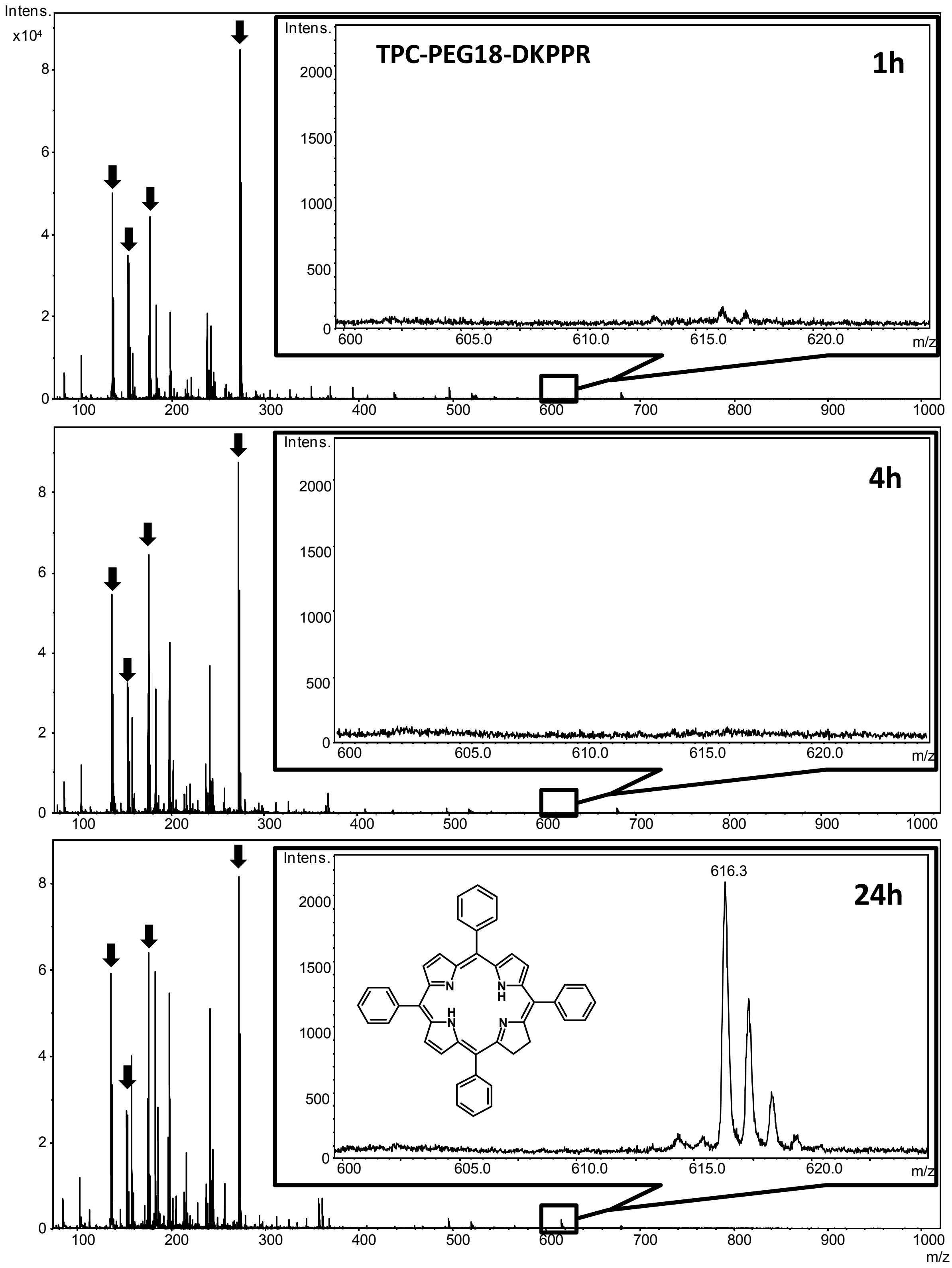

Figure S4. MALDI-TOFMS spectra of TPC–PEG18–DKPPR obtained with plasma samples after 1, 4 and 24 h post administration. Mass values are given with only one significant digit to avoid overloading the figure. Black arrows are corresponding to mass peaks belonging to the matrix.

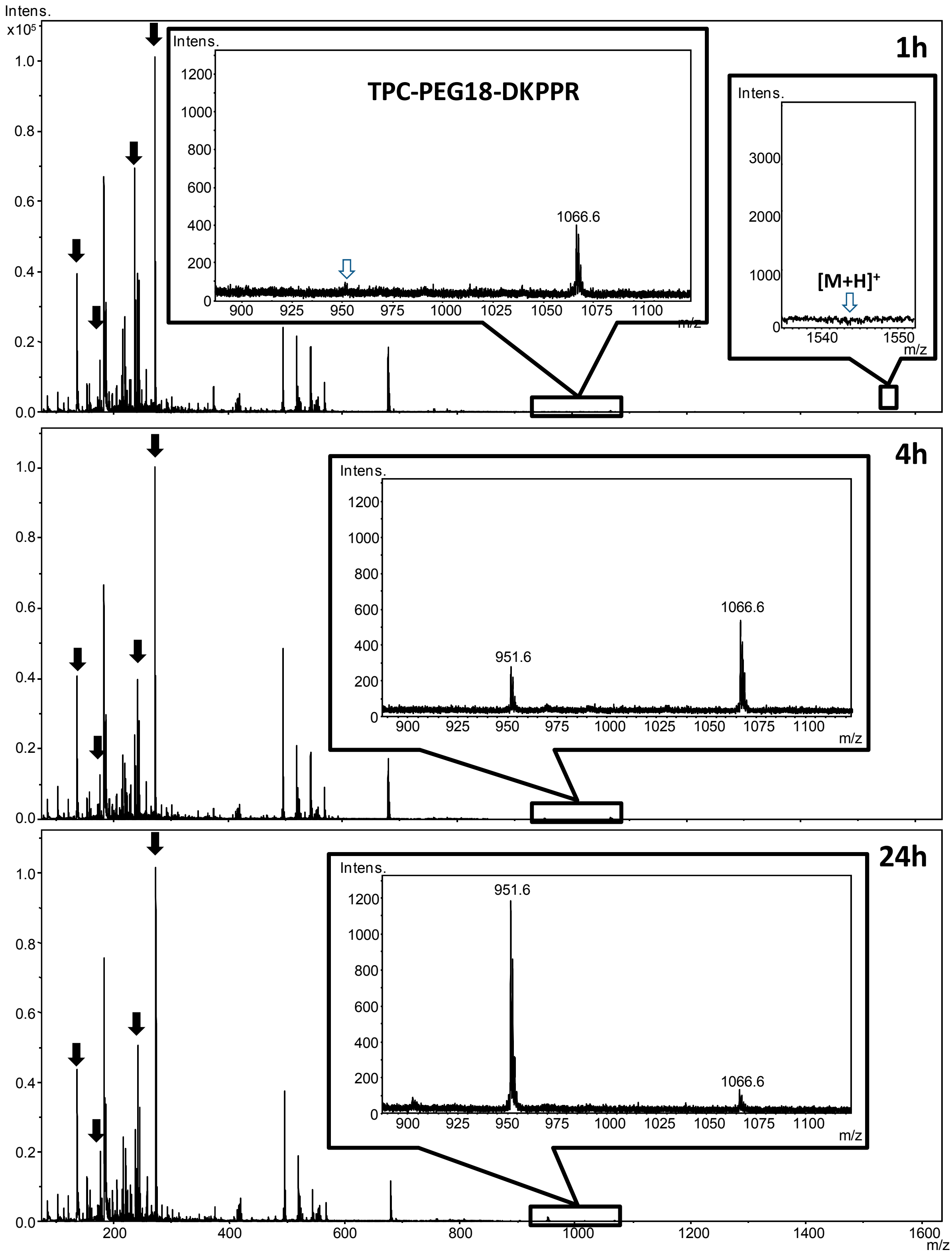

Figure S5. MALDI-TOFMS spectra of TPC–PEG18–DKPPR obtained with liver samples after 1, 4 and 24 h post administration. Mass values are given with only one significant digit to avoid overloading the figure. White arrows are corresponding to expected mass peaks. Black arrows are corresponding to mass peaks belonging to the matrix.

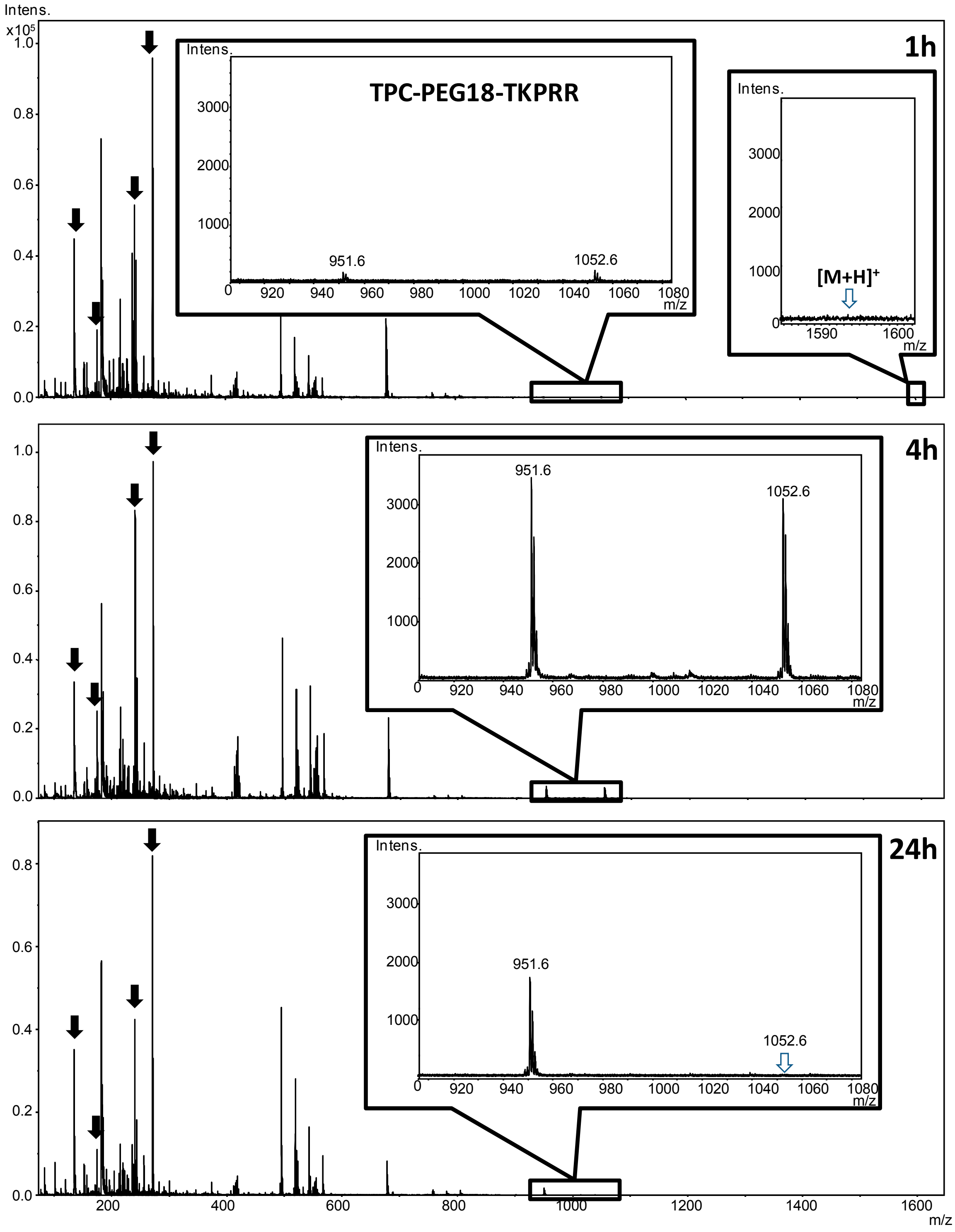

Figure S6. MALDI-TOFMS spectra of TPC–PEG18–TKPRR obtained with liver samples after 1, 4 and 24 h post administration. Mass values are given with only one significant digit to avoid overloading the figure. White arrows are corresponding to expected mass peaks. Black arrows are corresponding to mass peaks belonging to the matrix.
